# THE LANGUAGE OF LONELINESS: ANALYZING UNSTRUCTURED SPEECH TO DETECT LONELINESS AND SOCIAL ISOLATION

**DOI:** 10.1093/geroni/igae098.1881

**Published:** 2024-12-31

**Authors:** Ellen Lee, Ning Wang, varsha Badal, Sanchit Goel, Koduvayur Subbalakshmi

**Affiliations:** University of California San Diego, La Jolla, California, United States; Jiangnan University, Wuxi, Jiangsu, China (People’s Republic); University of California San Diego, La Jolla, California, United States; University of California San Diego, La Jolla, California, United States; Stevens Institute of technology, Hoboken, New Jersey, United States

## Abstract

The mental and physical toll of loneliness and social isolation lead to increased mortality for older adults. Early detection of social disconnection will aid in delivering timely interventions, however current assessments involve long surveys that cannot capture the individualized experiences of loneliness. Our group has been analyzing unstructured speech data using Natural Language Processing (NLP)and openSMILE as well as identifying linguistic and audio features that are associated with social disconnection among older adults. We will present our findings from using linguistic features alone, thematic categories, analyses of different interview segments, sex differences, and audio features. We will also present the association of social disconnection with longitudinal physical, mental, and cognitive outcomes in a community-dwelling healthy aging sample in San Diego. The sample includes older adults living independently in senior housing communities who have completed semi-structured interviews, sleep and physical activity assessments using wearable fitness trackers, a cognitive assessment battery, and clinical assessments for mental health concerns.

